# When Resuscitation Meets Reality: Catastrophic Type A Aortic Dissection Presenting as Trauma and the Emergency Physician’s Role Beyond Resuscitation

**DOI:** 10.51894/001c.165994

**Published:** 2026-07-31

**Authors:** Jasmine Philipose, Seynab Farah, Jash Mody, Abe Al-Hage

**Affiliations:** 1 Henry Ford - Wyandotte

## 112

### Introduction

Acute Stanford type A aortic dissection (ATAAD) is a life-threatening condition with mortality increasing by approximately 1–2% per hour without surgical intervention. While sudden severe chest pain is the classic presentation, atypical manifestations such as syncope, trauma, and altered mental status (AMS) can obscure diagnosis. These presentations may trigger trauma activation, delaying recognition of the underlying vascular catastrophe. In cases with severe neurologic injury and hemodynamic instability, emergency physicians may face the dual responsibility of aggressive resuscitation and early goals-of-care discussions.

### Case Presentation

A 72-year-old female with multiple comorbidities presented after a fall of unclear mechanism and was activated as a red-level trauma due to profound AMS with Glasgow Coma Scale (GCS) 3. Initial vitals: BP 135/104 mmHg, HR 75 bpm, RR 26/min, O₂ saturation 70% on non-rebreather. She was unresponsive and emergently intubated. Initial CT head revealed hypodensity in the left MCA territory concerning for acute stroke. CTA head and neck demonstrated dissection extending into the bilateral common carotid arteries with probable distal left A2 thrombus. Subsequent CTA chest, abdomen, and pelvis revealed an extensive Stanford type A aortic dissection from the aortic root through the thoracic aorta into the proximal left common iliac artery, involving the left subclavian and axillary arteries. Following imaging, she developed profound hypotension (60/40 mmHg) and required aggressive resuscitation including IV fluids, massive transfusion protocol, and vasoactive infusions (phenylephrine, esmolol). She was transferred for emergent cardiothoracic surgery. Despite intervention, her condition deteriorated, and the family elected do-not-resuscitate (DNR) status in the ICU.

### Discussion

Syncope occurs in 13–19% of ATAAD cases and is linked to worse outcomes. In this patient, extensive vascular involvement and cerebral malperfusion caused stroke and profound neurologic impairment. The combination of GCS 3, multi-arterial Type A dissection, hemodynamic instability requiring massive interventions, and large-vessel stroke indicated an extremely poor prognosis despite rapid diagnosis and transfer for definitive surgical care. While emergency physicians are trained to pursue aggressive life-saving measures, catastrophic presentations may warrant early consideration of prognosis and goals-of-care. In such scenarios, emergency physicians serve not only as resuscitationists but also as facilitators of compassionate, patient-centered end-of-life decision-making.

